# In time: vitamin D deficiency: who needs supplementation?

**DOI:** 10.1016/j.rppede.2015.10.003

**Published:** 2016

**Authors:** Tania Winzenberg, Graeme Jones

**Affiliations:** a Menzies Institute for Medical Research, University of Tasmania, Hobart, Tasmania, Australia; b Faculty of Health, University of Tasmania, Hobart, Tasmania, Australia

Vitamin D has a critical role in calcium metabolism and bone health in children and is postulated to contribute to non-bone conditions, such as respiratory illnesses, atopy and schizophrenia.^1^ There are clear links between vitamin D deficiency and rickets and neonatal hypocalcaemia, but vitamin D also has a potential role in optimising bone acquisition in childhood.^2^ Thus the need for vitamin D supplements requires consideration from a range of perspectives.

## Rickets

Rickets can be caused by low serum vitamin D levels and/or low dietary calcium intakes, and, less commonly, by disorders of phosphate metabolism. It is important to differentiate these causes as vitamin D supplements alone will not correct rickets unless vitamin D deficiency is the only or major cause. This is seen in a randomised control trial (RCT) in Nigerian children with rickets in which only 19% of those treated with vitamin D alone had near complete resolution, compared to 61% and 58% of those treated with calcium and calcium with vitamin D, respectively.^3^ In developing countries calciopenic rickets appears more common. Vitamin D given with calcium for calciopenic rickets can result in better outcomes that calcium alone.^4^

When low calcium intake contributes to rickets, causation may be incorrectly attributed to vitamin D levels which can result in overestimation of a threshold of vitamin D above which vitamin D deficiency rickets does not occur. However, in a RCT of vitamin D supplementation for rickets prevention in Chinese infants, even with low serum 25-hydroxy vitamin D (25(OH)D) concentrations (around 30nmol/L) rickets did not occur.^5^ Thus vitamin D-related rickets may be most common below this level. This is consistent with data from developed countries where rickets predominantly occurs in populations at known high risk of moderate to severe vitamin D deficiency such as African American populations in the US^6^ and dark-skinned immigrant populations in Australia.^7^^,^ 8 In the latter studies, serum 25(OH)D was <20mmol/L in 73% of cases and in 88% of cases aged less than 6 months,^7^ though rickets occurred at up to 50nmol/L.^8^ Therefore, in developed countries, these high risk groups require intervention, either by screening for and correcting significant vitamin D deficiency or by routine vitamin D supplementation of breastfeeding infants at high risk.

## Vitamin D supplementation to optimise peak bone mass

Observational evidence links vitamin deficiency in utero and childhood to reduced bone mineral density,^2^ but RCT data are limited. There are no RCTs of vitamin D supplementation in pregnancy with bone density outcomes in children. In general human milk-fed infants have lower bone accretion compared to formula fed infants.^9^ While this deficit appears temporary, as catch up growth occurs, it is possible that bone development in breastfeeding infants could be augmented through vitamin D supplements. This and concerns about the risk of rickets in children at risk of vitamin D deficiency has led to widespread recommendation of vitamin D supplementation of breastfeeding infants.^2^ Unfortunately, RCT data are sparse and unconvincing. Of three small trials of 400IU daily of vitamin D, none demonstrated any benefits of vitamin D supplementation on bone density in the first year of life.^2^ However, more than half the infants were likely to have vitamin D levels >50nmol/L, so RCTs in deficient infants are urgently needed before benefits in such infants can be ruled out. This is important as vitamin D supplements may only benefit bone mass in deficient children. In a meta-analysis^10^^,^ 11 of six RCTs, vitamin D supplementation had no statistically significant or clinically important effects on total body bone mineral content (TB BMC), hip bone mineral density (BMD) or forearm BMD, with a trend to a small effect on lumbar spine (LS) BMD (standardised mean difference (SMD)+0.15, (95%CI −0.01 to +0.31), *p*=0.07), when studies were analysed regardless of mean study baseline 25(OH)D. However, when grouped by mean baseline 25(OH)D, there were statistically significant effects on TB BMC and LS BMD in studies with mean baseline 25(OH)D <35nmol/L, and the magnitude of effects at all sites were at least 0.2 SMD higher than in studies with mean baseline 25(OH)D ≥35nmol/L. Even in studies with mean serum 25(OH)D <35nmol/L, around 20% of children would be vitamin D replete, so to properly estimate the magnitude of any benefits, RCTs targeting deficient children are needed.

## Vitamin D and other chronic diseases

The suggestion that vitamin D levels in childhood are related to the occurrence of other chronic diseases is based on limited observational data,^1^ with only sparse confirmatory RCT evidence. The exception is for birth weight, where observational and RCT data are congruent with a pooled effect size in RCTs of 130g.^12^ Despite observational associations with respiratory illnesses, RCTs demonstrate no benefit of vitamin D supplements for prevention of pneumonia in infants, or of maternal supplementation in pregnancy for risk of wheeze in offspring at 3 years of age.^1^^,^ 13 In infants with pneumonia from a population at high risk of deficiency, vitamin D did not reduce illness duration but reduced the likelihood of repeat pneumonia within 90 days (RR 0.78, 95%CI 0.64–0.94).^14^

## Conclusion

In summary, vitamin D supplementation of pregnant women or children at high risk of very low serum 25(OH) levels is clearly required to prevent rickets and neonatal hypocalcaemia. Supplementation is also needed to treat children with vitamin D deficiency rickets and potentially augment calcium supplements in calciopenic rickets. The ability to improve peak bone mass by correcting vitamin D deficiency in children or in pregnancy remains unproven, so routine vitamin D supplementation cannot be recommended for this purpose. Evidence that vitamin D supplementation improves other health-outcomes is also insufficient to support widespread supplementation.
