# Supplementary material for: In time: vitamin D deficiency: who needs supplementation?
Source: Rev Paul Pediatr. 2016 Jan-Mar;34(1):3–4. doi: 10.1016/j.rppede.2015.10.003 (PMC4795714; doi:10.1016/j.rppede.2015.10.003)
Supplement: Translation

# Translation: Em tempo: deficiência da Vitamina D: quem precisa de suplementação?

**Authors:** Tania Winzenberg, Graeme Jones

**Affiliations:** a Instituto Menzies de Pesquisa Médica, Universidade da Tasmânia, Hobart, Tasmânia, Austrália; b Faculdade de Saúde, Universidade da Tasmânia, Hobart, Tasmânia, Austrália

A vitamina D tem um papel fundamental no metabolismo de cálcio e na saúde óssea das crianças. Acredita-se ainda que ela seja benéfica em doenças não ósseas, tais como doenças respiratórias, atopia e esquizofrenia.^1^ Existe uma relação clara entre a deficiência de vitamina D e raquitismo e hipocalcemia neonatal, mas a vitamina D também facilita a aquisição de massa óssea na infância.^2^ Assim, a necessidade de suplementação dessa vitamina precisa ser considerada sob uma série de perspectivas.

## Raquitismo

O raquitismo pode ser causado por baixos níveis séricos de vitamina D e/ou por ingestão dietética de cálcio insuficiente; e, menos comumente, por distúrbios no metabolismo de fosfato. É importante diferenciar essas causas, já que a suplementação de vitamina D de forma isolada não cura o raquitismo, a menos que essa deficiência seja a causa única ou principal da doença. Isso foi observado em um estudo clínico randomizado (ECR) com crianças nigerianas com raquitismo, no qual apenas 19% daquelas tratadas exclusivamente com vitamina D tiveram resolução quase completa do quadro, comparadas com 61% e 58% das tratadas com cálcio e cálcio e vitamina D, respectivamente.^3^ O raquitismo hipocalcêmico é mais comumente observado em países em desenvolvimento. O uso de vitamina D combinada com cálcio para o tratamento de raquitismo hipocalcêmico traz resultados melhores do que o uso de cálcio isoladamente.^4^

Quando a baixa ingestão de cálcio contribui para o raquitismo, o nexo de causalidade pode ser incorretamente atribuído aos níveis de vitamina D, o que resulta na superestimativa do limiar de vitamina D, acima do qual o raquitismo por deficiência de vitamina D não ocorreria. No entanto, em um ECR que analisou a suplementação dessa vitamina para a prevenção de raquitismo em crianças chinesas, mesmo com baixas concentrações séricas de 25-hidroxi-vitamina D (25[OH]D; cerca de 30nmol/L), a doença não foi observada.^5^ Assim, o raquitismo dependente de vitamina D pode ser mais comum abaixo desse nível. Isso é consistente com os dados de países desenvolvidos, nos quais o raquitismo ocorre predominantemente em populações sabidamente em alto risco para deficiência de vitamina D moderada a grave, tais como populações Afro-Americanas – nos Estados Unidos^6^ – e populações de imigrantes de pele escura – na Austrália.^7^^,^ 8 Em estudos mais recentes, observaram-se níveis séricos de 25(OH)D<20mmol/L em 73% dos casos e em 88% dos casos com idade inferior a seis meses,^7^ embora raquitismo tenha sido observado em pacientes com até 50nmol/L.^8^ Por isso, nos países desenvolvidos é necessária a intervenção nesses grupos de alto risco, seja por triagem e correção deficiências significativas de vitamina D seja por suplementação de vitamina D de rotina em lactentes de alto risco.

## Suplementação de vitamina D para aumentar o pico de massa óssea

Evidências observacionais relacionam a deficiência de vitamina no útero e na infância à redução da densidade mineral óssea em crianças,^2^ mas os dados de ECRs são limitados. Nenhum ECR abordou a suplementação de vitamina D na gravidez e sua associação com resultados de densidade óssea em crianças. De forma geral, lactentes amamentados têm menor crescimento ósseo em comparação com aqueles alimentados com fórmula.^9^ Embora esse déficit seja aparentemente temporário, já que observa-se a recuperação do crescimento, é possível que o desenvolvimento ósseo em crianças amamentadas possa ser aumentado com suplementação de vitamina D. Essa premissa e as preocupações com o risco de raquitismo em crianças em risco de deficiência de vitamina D levaram à recomendação generalizada de suplementação de vitamina D para lactentes.^2^ Infelizmente, os dados de ECRs são escassos e pouco convincentes. De três pequenos ensaios com suplementação diária de 400UI de vitamina D, nenhum demonstrou quaisquer benefícios da suplementação de vitamina D na densidade óssea no primeiro ano de vida.^2^ No entanto, mais da metade das crianças estudadas apresentavam níveis de vitamina D provavelmente superiores a 50nmol/L; portanto, ECRs com crianças com deficiência de vitamina D são necessários urgentemente antes que os benefícios de sua suplementação para tais crianças possam ser descartados. Isso é importante, já que é possível que a suplementação de vitamina D seja benéfica à massa óssea apenas em crianças deficientes. Em uma metanálise^10^^,^ 11 de seis ECRs, a suplementação de vitamina D não teve efeitos estatisticamente significantes ou clinicamente importantes sobre o conteúdo mineral ósseo total do corpo (CMO-TC) nem sobre a densidade mineral óssea (DMO) do quadril ou antebraço, mas apresentou uma tendência para um pequeno efeito sobre a DMO da coluna lombar (DMO-CL; diferença média padronizada [DMP] +0,15; [IC95%: 0,01 a 0,31]; *p* = 0,07), quando os estudos foram analisados independentemente da média inicial de 25(OH)D. No entanto, quando agrupados por média inicial de 25(OH)D, foram observados efeitos estatisticamente significantes no CMO-TC e na DMO-CL em estudos com média inicial de 25(OH)D <35 nmol/L. A magnitude dos efeitos em todos os locais foi no mínimo 0,2 DMP maior do que em estudos com média inicial de 25(OH)D ≥35nmol/L. Mesmo em estudos com média de níveis séricos de 25(OH)D <35 nmol/L, cerca de 20% das crianças teriam níveis de vitamina D adequados. Portanto, para estimar corretamente a magnitude de quaisquer benefícios, são necessários ECRs dirigidos às crianças deficientes.

## A vitamina D e outras doenças crônicas

A sugestão de que os níveis de vitamina D na infância estejam relacionados com a ocorrência de outras doenças crônicas é baseada em dados observacionais limitados,^1^ com poucas evidências de ECR confirmatórias. A exceção é o peso ao nascer, no qual os dados observacionais e ECR são congruentes com um tamanho de efeito agrupado entre ECRs de 130g.^12^ Ainda que existam associações observacionais com doenças respiratórias, ECRs não demonstraram benefício da suplementação de vitamina D na prevenção da pneumonia em crianças ou da suplementação materna durante a gestação no risco de chiado na criança aos três anos de idade.^1^^,^ 13 Em crianças com pneumonia provenientes de uma população em alto risco de deficiência, a vitamina D não reduziu a duração da doença, mas diminuiu a probabilidade de recorrência dentro de 90 dias (RR: 0,78; IC95%: 0,64-0,94).^14^

## Conclusão

A suplementação de vitamina D em grávidas ou em crianças com alto risco para níveis séricos de 25(OH) muito baixos é claramente necessária para evitar o raquitismo e a hipocalcemia neonatal. A suplementação também é necessária para o tratamento de crianças com raquitismo associado à deficiência de vitamina D e, potencialmente, para reforçar os suplementos de cálcio no raquitismo hipocalcêmico. Não existe comprovação de que o pico de massa óssea pode ser aumentado com a correção da deficiência de vitamina D em crianças ou na gestação. Portanto, a suplementação rotineira dessa vitamina não pode ser recomendada para essa finalidade. A evidência de que a suplementação de vitamina D melhora outros desfechos de saúde também é insuficiente para fundamentar a suplementação generalizada.
